# OS2: Oblivious similarity based searching for encrypted data outsourced to an untrusted domain

**DOI:** 10.1371/journal.pone.0179720

**Published:** 2017-07-10

**Authors:** Zeeshan Pervez, Mahmood Ahmad, Asad Masood Khattak, Naeem Ramzan, Wajahat Ali Khan

**Affiliations:** 1 School of Engineering and Computing, University of the West of Scotland, Paisley, PA1 2BE, United Kingdom; 2 Ubiquitous Computing Lab, Department of Computer Engineering, Kyung Hee University, Global Campus, 1 Seocheon-dong, Giheung-gu, Yongin-si, Gyeonggi-do 446-701, South Korea; 3 College of Technological Innovation, Zayed University, Abu Dhabi Campus, United Arab Emirates; University of Texas at San Antonio, UNITED STATES

## Abstract

Public cloud storage services are becoming prevalent and myriad data sharing, archiving and collaborative services have emerged which harness the pay-as-you-go business model of public cloud. To ensure privacy and confidentiality often encrypted data is outsourced to such services, which further complicates the process of accessing relevant data by using search queries. Search over encrypted data schemes solve this problem by exploiting cryptographic primitives and secure indexing to identify outsourced data that satisfy the search criteria. Almost all of these schemes rely on exact matching between the encrypted data and search criteria. A few schemes which extend the notion of exact matching to similarity based search, lack realism as those schemes rely on trusted third parties or due to increase storage and computational complexity. In this paper we propose Oblivious Similarity based Search (OS2) for encrypted data. It enables authorized users to model their own encrypted search queries which are resilient to typographical errors. Unlike conventional methodologies, OS2 ranks the search results by using similarity measure offering a better search experience than exact matching. It utilizes encrypted bloom filter and probabilistic homomorphic encryption to enable authorized users to access relevant data without revealing results of search query evaluation process to the untrusted cloud service provider. Encrypted bloom filter based search enables OS2 to reduce search space to potentially relevant encrypted data avoiding unnecessary computation on public cloud. The efficacy of OS2 is evaluated on Google App Engine for various bloom filter lengths on different cloud configurations.

## 1 Introduction

We are living through an era of data intensive applications in which digital data is doubling almost every eighteen months [1]. With the emergence of Big data, companies ranging from small to large scale enterprises are trying to make most out of the data gathered from their customers and business processes [2]. Data ranging from sharable (social network data) to confidential (personal healthcare records) in nature are processed and analyzed by tools and technologies which enable Big data [3], [4]. In the context of data management, cloud based storage services are becoming prevalent as these services offer cost effective solutions to persist, process and provision large amount of data following the notion of pay-as-you-go business model. With virtualized and on-demand provisioning of cloud infrastructure (i.e., networking facility, computation power, and storage capacity) these services enable their subscribers to scale storage and computational facilities according to their requirements.

Since, these services are offered through untrusted cloud service providers there is a great risk of privacy infringement when personal and confidential data are outsourced to such services [5], [6], [7]. Personal health records, financial statements and business plans are few examples of sensitive data which can seriously affects the lives of individuals and businesses, if compromised. The most obvious solution to ensure data privacy is to always outsource data in encrypted form and share the corresponding decryption keys with authorized users to whom data is shared [8], [9]. Although encrypted data (we refer outsourced data as encrypted data, and throughout the text in subsequent sections they are used interchangeably) restrains cloud server provider from compromising privacy of the data; however, it significantly reduces the capabilities of a user to access relevant data by using conventional search queries [10], [11]. Besides this, the scope of privacy related issues are not limited to the outsourced data only, cloud service provider can use deductive reasoning to learn private and confidential information about the data owner i.e., if outsourced clinical reports of a user are accessed by a medical doctor specialized in diabetes mellitus, cloud service provider can deduce that there is a possibility that user is a diabetic patient.

Cloud service providers charge their subscribers (users) according to the magnitude of service usage i.e., network, storage and processing [12], [13]. To ensure efficient utilization of cloud infrastructure it is very important for subscribers of a cloud storage to access only relevant data. Since, outsourced data is in encrypted form conventional search queries cannot be used to identify relevance between the outsourced data and search criteria. However, to solve the problem of searching encrypted data sizable number of systems and algorithms have been proposed which are generally referred as search over encrypted data schemes [14]. These schemes either exploit the cryptographic primitives or indexing methodologies to search outsourced data. Schemes that primarily focus on cryptography utilize trapdoors defined over the encrypted data—a trapdoor is defined for a particular keyword and is shared with authorized subscribers to search outsourced data [15], [16]. Whereas, indexing based schemes utilize keyword extraction algorithms to identify important words (index) from the outsourced data and then store them either in a trusted domain (trusted third party) or in semi-trusted domain where it cannot be linked with the outsourced data i.e., semi-trusted entity persisting index does not collude with the cloud service provider provisioning the outsourced data [17], [18], [19].

So far, search over encrypted data schemes have focused on ensuring privacy of the data and search queries. For search query evaluation these schemes mainly consider exact matching between the outsourced data and search criteria. Consequently, these schemes are only able to retrieve outsourced data where there is an exact match between the trapdoor and outsourced data (trapdoor based cryptography [15], [16]) or search criteria and index computed from the outsourced data (index based encrypted data search [20]). This notion of exact matching is completely different than what is used in real world to search data over the internet and for querying conventional database i.e., identifying similarity between the data and search criteria. Thus, a search scheme which can provide similarity based search over encrypted data would greatly elevate the search experience of cloud storage subscribers by assisting them in accessing relevant outsourced data even if search criteria is marginally erroneous to be matched with the outsource data i.e., typographical errors or misspelled keywords.

A few schemes have been proposed focusing on similarity based searching for encrypted data. These schemes either rely on edit distance based measures to realize encrypted search queries which are resilient to typographical errors [21] or employ secure probabilistic dimension reduction to measure the similarity between the outsourced data and search criteria [10]. Although these schemes realize privacy-aware data search which do not reveal any information about the outsourced data and search criteria; however, malicious query evaluator (cloud service provider) can still learn the result of query evaluation process i.e., relevance measure between the outsourced data and search criteria. Result of query evaluation process can be exploited by employing deductive reasoning (as described earlier) to passively compromise privacy of the outsourced data and data owner as well. Besides the passive attack, these schemes mainly rely on assumptions which either do not align with the real world or are computationally infeasible. Pre-computing all possible typographical errors of a word would greatly effect the computational load and storage capacity as query evaluator would have to match search criteria with every possible pre-computed encrypted typographical error. Secure probabilistic dimension reduction rely on engaging two cloud service providers, one for persisting outsourced data and second for evaluating encrypted search queries.

To realize privacy-aware relevant data access by using encrypted search queries in this paper we propose oblivious similarity based searching for encrypted data (OS2). Unlike conventional search over encrypted data, OS2 realizes similarity based search which utilize a real-valued function quantifying the similarity between the outsourced data and search criteria instead of merely stating binary values i.e., matched or unmatched. Besides this, OS2 restrains malicious cloud service provider to passively compromise privacy of the outsourced data and data owner, by learning the result of search query evaluation. In contrast with conventional search over encrypted data methodologies, OS2 does not rely on trusted or semi-trusted third parties to process encrypted search queries. Basic building blocks of OS2 are encrypted bloom filter [22] constructed from n-grams and probabilistic homomorphic encryption [23]. These building blocks ensure end-to-end privacy-aware search for cloud based storage services without relying on trusted or semi-trusted third party to process search queries.

### 1.1 Main idea

The main idea of OS2 can be explained using following realistic scenario in which multiple users are collaborating over confidential data, outsourced to an untrusted cloud service provider in an encrypted form.

Suppose Alice is a neurosurgeon working in a national hospital. She is an expert in acute neurological problems and treats patients suffering from neurological disorders. She is also actively involved in clinical research to discover new medicines and their effects on patients. With the consent of her patients she complies a comprehensive report for each of her patients. Her assistant Bob is responsible for meticulously compiling the reports, which include daily clinical and non-clinical observations and medical history over the period of treatment.

Mallory is a senior research fellow at a medical research institute. She is interested in conducting a comprehensive study on Alzheimers and Parkinsons diseases. For that she is collaborating with Alice with an understanding that Alice will share reports of her patients (reports compiled by Bob) and Mallory will share her clinical findings. To efficiently collaborate and share data both Alice and Mallory subscribe to a cloud based storage service managed by Eve.

To ensure data confidentiality Alice and Mallory have decided to outsource encrypted data, and share necessary cryptographic primitives and keys. Since, Eve charges her subscribers on amount of data consumed on each data access request, an encrypted data structure is also outsourced to Eve’s cloud. Encrypted data structure is designed to ensure that search results are resilient to typographical errors and results can be ranked according to their relevance to the search criteria.

Alice and Mallory search the outsourced data by using a search criteria transformed to an encrypted search query before submitting to Eve. Encrypted search query is used to learn the similarity between the encrypted data structure and search criteria. The entire process of search query evaluation is executed by Eve; however, its result is oblivious to her. This ensures that Eve cannot learn any useful information from the search results, which can lead to potential loss of data privacy. Since, only Alice and Mallory have exchanged necessary cryptographic keys, a malicious subscriber colluded with Eve cannot query encrypted data structure successfully.

### 1.2 Contributions

In this paper with OS2 we make the following contributions in the area of search over encrypted data within the domain of untrusted cloud based storage services:

- Bloom filter based oblivious search for encrypted data, which can evaluate a real-valued similarity function to measure relevance between the outsourced data and search criteria.
- Reduced number of unnecessary comparison operations between the outsourced data and search criteria. Auxiliary information about the bloom filter bit locations is utilized to minimize the search space.
- Oblivious search evaluation without relying on any trusted or semi-trusted third party which ensures efficient utilization of cloud infrastructure. Oblivious evaluation of search query restrains cloud service provider from passively deducing confidential information which cannot be learned from the outsourced data otherwise.

It is worth mentioning that Eu-Jin Goh proposed first bloom filter based search [24]. It used trapdoors defined for specific keywords to retrieve matching documents i.e., exact match between the trapdoor and bloom filters (document indexes). However, the contribution of OS2 is the novel use of sliding window (please refer to Section 4) with bloom filters to evaluate relevance based outsourced data and search criteria.

The rest of the paper is organized as follows: Section 2 reviews the related work in the area of encrypted data search for untusted domains. Section 3 presents the system models, design goals and assumptions. Section 4 describes the proposed methodology of oblivious similarity based search (OS2). Section 5 explains the implementation details of similarity based search for untrusted cloud service provider. Section 6 presents the evaluation results of OS2 on Google App Engine. Section 7 presents the security analysis of OS2. Section 8 concludes the paper along with future directions in the context of oblivious similarity based search for encrypted data.

## 2 Related work

We categorize OS2 as a secure content discovery service within in an untrusted domain rather than a new cryptosystem which provides trapdoor based search for encrypted data. OS2 leverages subscribers of a public cloud based storage service to obliviously learn relevance between their defined search queries and outsourced data. This section presents existing schemes to search encrypted data, some of them exploit the cryptographic primitives; whereas, others focus on secure indexes to exactly match search criteria with the outsourced data. We mainly focus on efficacy of these schemes to retrieve relevant outsourced data and involvement of external entities to ensure privacy. We also examine the possibility of a passive attacks if untrusted entity (cloud service provider) learns result of a search query.

Searchable encryption based on symmetric encryption called searchable symmetric key cryptography (SKC) was first proposed by Song et al., [15]. SKC defines a trapdoor for a particular keyword which is then used to learn exact matching between the trapdoor and encrypted data. Based on SKC several schemes have been proposed which utilize trapdoor based encryption to search encrypted index, instead of the data [25, 26, 27]. Similar to SKC, public key cryptography (PKC) was first proposed by Boneh et al., [16]. PKC enables trapdoor evaluation for data encrypted with asymmetric encryption. Both SKC and PKC rely on trapdoor evaluation function which can only learn exact matching between the search criteria (trapdoor) and encrypted data. In the context of public cloud based data sharing services these schemes require exchange of trapdoors between the data owner and authorized subscribers. Besides this, each trapdoor is defined for a particular keyword only, it greatly effects the searching capability of authorized subscribers as they can only search encrypted data for limited number of keywords.

Authorized private keyword search (APKS) over encrypted personal records was proposed by Li et al., [17]. In their proposed scheme they utilize Trusted Third Party (TTP) to distribute capabilities (trapdoors) to authorized subscribers according to their access privileges. These capabilities are then used to learn exact matching between the trapdoors and personal health records. Similar to SKC and PKC, APKS offers a limited search experience as authorized subscribers can only search for those keywords for which trapdoors are defined by the data owner. Wang et al., [28] proposed a trapdoor based relevance search over encrypted data persisted in an untrusted domain. However, their scheme is only limited to a single trapdoor based search query. Thus, lacking realism for searching relatively huge amount of data where there is a need to learn relevance according to multiple search criterion.

Searchable cryptographic cloud storage system (CS2) proposed search over encrypted data focusing dynamic updates of the outsourced data [29]. CS2 search encrypted data by evaluating an exact matching function between encrypted inverted index and search criteria. However, CS2 is only confined to personal cloud based storage service and is not applied for cloud-based data sharing and collaboration services. Similary, [30] proposed a secure and efficient update scheme for encrypted data search. As with the other schemes, [30] performed search operations over the encrypted index; however, the proposed scheme was only confined to search query evaluation using binary operation of matched and unmatched search criteria—it cannot be extended to learn relevance between search query and encrypted index.

To incorporate multiple keyword search over encrypted data, Wenhai Sun et al., [31] proposed a privacy-preserving multi-keyword text search (MTS) with similarity-based ranking. MTS utilizes tree-based indexing with adaption methods for a multi-dimensional algorithm. Although MTS ensures privacy of search criteria and tree based index; however, it is based on an assumption that subscriber searching the cloud storage always behaves honestly and cloud server provider is honest-but-curious. Clearly, this assumption lack realism in the context of public cloud based storage services which leverage subscribers to share and collaborate on outsourced data—an unauthorized subscriber can behave maliciously to learn presence of a particular keyword to deduce personal / confidential information which cannot be learned otherwise. Similarly, [32] also provided multi-keyword ranked search over encrypted cloud data using same assumption of honest-but-curious model. Oblivious Term Matching (OTM) proposed an encrypted index oblivious search, where the index is computed over encrypted outsourced data [33]. OTM obliviously evaluates conjunctive search queries, thus enabling authorized subscribers to define complex selection criterion based on multiple keywords. Oblivious evaluation of search queries retrain untrusted cloud service provider from deducing personal / confidential information which can lead to potential loss of privacy. However, OTM evaluates exact matching function between a conjunctive search query and encrypted index entries. In [34] authors proposed searchable symmetric encryption with conjunctive queries focusing on scalability issues of encrypted data search. Similarly to others, the scheme did not support relevance based search queries.

To overcome the limitations of exact matching between search criteria and outsourced data Mehmet et al., [10] proposed efficient similarity search over encrypted data. To find relevance between the search criteria and encrypted data they utilize a fast nearest neighbor search in high dimensional space called locality sensitive hashing (LSH) [35]. Their search over encrypted data scheme is based on secure index structure that is built through LSH, which maps index entries into several buckets such that similar entries are stored into same buckets whereas, dissimilar entries do not with high probability. Through rigorous security analysis the authors showed that the proposed scheme was secure under adaptive semantic security for searchable semantic encryption. However, to prevent cloud server from learning identifiers of the outsourced data having close relevance with the search criteria, the authors proposed two servers setting—where one server is responsible for persisting the outsourced data and second server is in charge of evaluating search queries. Two servers setting is based on an assumption that both servers do not collude with each other. This assumption seriously effects the practicality of the scheme when deployed to search confidential informational. Besides this, in a single server setting cloud server can successfully compromise privacy of the outsourced data by learning its relevance with search criteria.

Fuzzy keyword search [21] is another search over encrypted data scheme designed specificity to search encrypted data outsourced to a public cloud storage service. It increases user search experience by incorporating privacy-aware search queries which are resilient to typographical errors. It utilizes trapdoor based encryption to search encrypted index associated with the outsourced data. To realize search over encrypted data scheme which is resilient to typographical errors, it pre-compute all possible typographical errors of a keyword with a certain edit distance measure. Although the authors manage to address the typographical errors; however, they mainly rely on exact matching between the pre-computed typographical errors and trapdoors. Besides this, pre-computing all possible typographical errors can significant increase the index size and as it is directly proportional to the value of edit distance used to compute all possible misplaced keystrokes which many result in an error.

Jingwei Li et al., [36] proposed privacy-preserving data utilization in hybrid clouds—a privacy-aware data utilization (search and accessibility) service which can restrain unauthorized subscribers from consuming data outsourced to a public cloud. The authors utilize hybrid cloud architecture in which access control policies are enforced by the private cloud; whereas, public cloud is responsible of persisting the outsourced data. To highlight efficacy of their system, the authors demonstrated fuzzy keyword search over encrypted data. In their hybrid architecture fuzzy search queries are generated by the private cloud delegating computational load of query formulation from user’s end to the cloud infrastructure. However, this type of hybrid cloud configuration requires data management in private cloud thus obstructing migration to public cloud and maximized utilization of public cloud infrastructure. Besides this, their fuzzy keyword search mainly rely on pre-computing all possible typographical errors and learning exact matching with the trapdoors—thus offering a primitive level of search experience. In [37] authors proposed an other fuzzy search over encrypted data to noisy search queries. The authors defined a closeness function (i.e., close, near or far) to evaluate similarity between search query and outsourced data. Although considered to be a first significant effort to realize fuzzy search over encrypted data; however, the defined closeness function was very primitive and cannot be extended to a notion of relevance i.e., matched and unmatched number of characters. Besides this, the scheme required large ciphertext in order to achieve fuzzy behavior in searchable encryption.

Considering the rapid adoption of cloud based storage services, enterprise wide search products like Google Search Appliance [18] and Microsoft Search Product [19] are leveraging their subscribers to search outsourced data. These specialized products can be used to query document repositories within the enterprise (private data centers) and public cloud based storage services as well. These products mainly rely on enterprise wide centralized index which is maintained within the enterprise’s data center. All search queries are evaluated for the centralized index and access control policies are enforced over the search queries to prevent unauthorized subscribers from querying the index. Since, search service is hosted by the enterprise itself, these search products greatly obstruct migration to cloud based storage services. Research work concluded in [38] has shown that by carefully modelling search queries malicious subscribers can learn valuable information from the centralized index, even if they do not have access to the data residing within private and public cloud based storage i.e., enterprise wide centralized index and outsourced data respectively.

Some recent developments in the area of search over encrypted data considered storage overhead, read-efficiency, capability of handle large databases, and verification of search results. In [39] authors constructed a searchable symmetric encryption scheme which provided optimal locality, space overhead, and nearly-optimal read efficiency—where locality was defined as maximum number of non-contiguous memory access that a server performed for each search request, and read efficiency as a ratio between the number of bits the server reads for each search request and the actual size of the answer. Distributed searchable symmetric encryption scheme for large scale database was proposed in [40]. The scheme constructed B-tree from a large scale database and performed encrypted search queries over the tree in two servers setting model. The main server (data owner) stored the large scale database; whereas, helper server was used to handle majority of the search queries. Verifiable searchable symmetric encryption scheme was proposed in [41] focusing on correctness and verifiability of the search results. The authors demonstrated that the proposed scheme could be easily extended to conjunctive and boolean queries.

**Fig 1:**
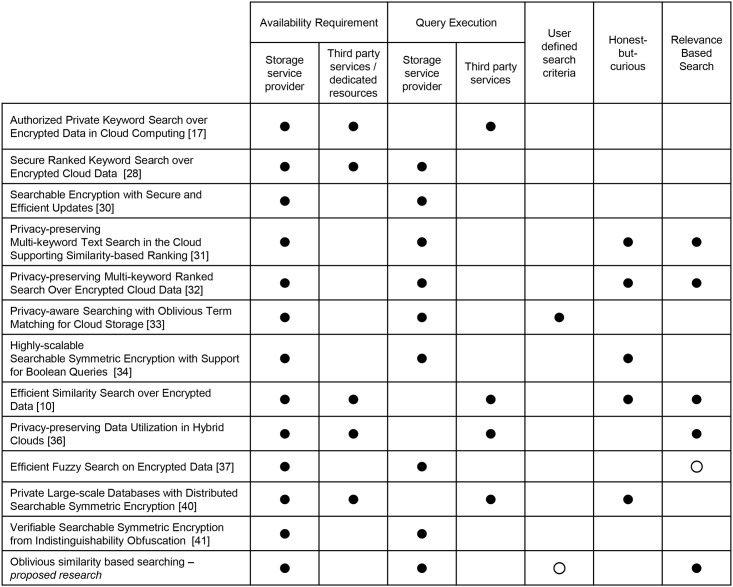
Comparative analysis of cloud based encrypted data search methodologies. Solid circle (●) represents availability requirement, entity responsible to evaluate search request, support for user defined search criteria and relevance based search, and reliance on honest-but-curious assumption. Hollow circle (∘) represents factors which can be supported by an extended version of the system.

In encrypted data search, factors like availability requirement of involved entities (cloud service provider, trusted/semi-trusted third party, or a dedicated server), entity responsible of evaluating server query, and ability to define search query (keywords) significantly affect the practicality of the system. For instance, a system relying on a third party to process search queries would impose availability requirement on a cloud service provider and third party. Availability of a cloud service provider can be reasonably achieved through service level agreements [42]; however for a third party, the system first needs to evaluate the trust and then keep track of all the interactions to ensure third party is not colluding with cloud server provider. Besides these factors, user’s ability to define its own search criteria, rather than simply relying on predefined trapdoors or encrypted search criteria and support for relevance based search query are also important for the realism of an encrypted data search. These factors are specifically important for achieving user experience which is close to normal search over plain text data. Assumption on the involvement of a cloud service provider and third party as honest-but-curious entities significantly affects the overall working on the encrypted data search. Fig 1 presents a comparative analysis of existing methodologies with our proposed oblivious similarity based searching (OS2). As OS2 does not rely on a trusted third party to evaluate search queries, it only requires availability of a cloud service provider which is well aligned with normal cloud service provisioning models. The main contribution of OS2 is relevance based search over encrypted data (more details in Section 4) which can be reasonably extended to support user defined search criteria without any substantial modifications to the proposed scheme.

## 3 System design and security goals and assumptions

### 3.1 System model

In a cloud based storage service scenario, we consider cloud service provider, data owner and data consumer as involved entities. For simplicity these entities are referred as cloud server, owner, and user respectively. Cloud server owns, manages and operates the cloud based storage service and provides access to its subscribers. Owner and user are subscribers of the cloud server. Owner manages a shared repository which is used to outsource encrypted data. User has access privileges on the shared repository. Since outsourced data is in encrypted form, user uses search query to identify and access only relevant data contents. Search query is evaluated by the cloud server and search results are sent back to the user.

### 3.2 Security model

For the proposed oblivious similarity based search (OS2) we adopted the notion of security in which any process performed on the data must not assist attacker(s) to deduce confidential information about the data. Privacy of the data outsourced to a cloud storage can be ensured by using cryptographic primitives. However, to access relevant data contents user must be able to obliviously search the data. The oblivious execution of search query ensures that cloud server cannot learn useful information by the query evaluation procedure, which can potentially compromise privacy of the outsourced data. To compromise privacy of the data, cloud server can collude with malicious users to learn the absence or presence of a particular keyword. They can also collude to learn the result of a particular search query submitted by an authorized user.

### 3.3 System design goals

The pivotal design goal of search over encrypted data is to enable subscribers to access relevant encrypted data without compromising privacy. Relevant access of data ensures that users can identify the encrypted which is most likely to contain the information they are searching for. A system identifying relevance between the encrypted data and search queries must be resilient to typographical error. Such a system will focus on similarity based matching instead of an exact match between the encrypted data and search query. Another important factor which must be considered is potential leakage of information which can be exploited by the cloud server and malicious users to compromise privacy of the data. Thus, a system providing encrypted data search should not reveal any information about the search results to the cloud server and malicious users. The entire processing of encrypted query evaluation should remain oblivious to the cloud server.

So, with OS2 we are realizing a privacy aware encrypted data search which can identify relevance between the encrypted data and concealed search queries. With these design goals, cloud server will be unable to learn any information from the query evaluation process which can be used to compromise privacy of the encrypted data and search query as well.

### 3.4 Assumptions and notations

Oblivious similarity based search (OS2) is specifically designed for public cloud based storage services. To ensure end to end privacy of the encrypted data and involved entities, we consider the cloud server as an untrusted entity. By untrusted entity, we mean that the cloud server tries to learn absence or presence of a particular word in the encrypted data by analysis results of search query. In order to search the encrypted data with privacy consideration, we assume that the cloud server executes oblivious similarity based search honestly. However, the cloud server can assist a malicious users to execute unauthorized search query to compromise privacy of the encrypted data and involved entities. For brevity we intentionally neglected the details of secure data sharing and only focused on privacy-aware relevant data access. Readers my refer to [43] for details on privacy-aware data sharing in public cloud. We assume that there exists an efficient indexing algorithm, which can extract important keywords from a file. To avoid compatibility issues while evaluating encrypted search queries we assume that size of bloom filters and family of hash function are predetermined between the owner and authorized users.

For the sake of simplicity in the descriptive detail of oblivious similarity based search (OS2), we use the notations shown in Table 1. F represents a file which is outsourced by the owner to a shared repository. I is an index which is computed from F, it contains list of important and frequently occurring keywords (*kw*_0_, *kw*_1_ … *kw*_*n*_). *π*_*i*_ is a bloom filter which is computed for *kw*_*i*_ by using a predetermined sliding window size. H is a family of hash functions which are used to set bit locations in *π*_*i*_ for each output of the sliding window over *kw*_*i*_. λ represents the size of a bloom filter. *τ*_*i*_ is a total number of bit positions which are set to *one* in *π*_*i*_. $B_{kw}$ is a data structure which contains 〈*π*_0_, *π*_1_ … *π*_*n*_〉 in encrypted form, along with the corresponding 〈*τ*_0_, *τ*_1_ … *τ*_*n*_〉. C is a search criteria containing list of search words (*sw*_0_, *sw*_1_ … *sw*_*j*_). *ρ*_*i*_ is a bloom filter which is computed for *sw*_*i*_ by using a predetermined sliding window size. Q is a search query submitted by a user, it contains 〈*ρ*_0_, *ρ*_1_ … *ρ*_*j*_〉 in encrypted form and a numeric value *ϕ* to filter $B_{kw}$. *ϕ* is a threshold value for identifying encrypted bloom filters which can produce higher value of similarity measure for encrypted search query evaluation. $E_{S}$, $D_{S}$ are symmetric encryption and decryption algorithms with a secert key *k*. $E_{H}$, $D_{H}$ are encryption and decryption algorithms from homomorphic cryptosystem, having (*σ*_*pk*_, *σ*_*sk*_) as public and secret key pair. ${\vec{\Delta }}_{0\ldots j\times m}$ is a result of oblivious search query evaluation.

**Table 1:** Notations used in the descriptive detail of OS2.

*Notation*	*Description*
F	File outsourced to a cloud based shared repository.
I	Index file consisting of *n* keywords *kw*_0_, *kw*_1_ … *kw*_*n*_.
*π*_*i*_	Bloom filter encoding $kw_{i}\in I$.
λ	Size of a bloom filter: total number of bit locations that can be marked as *zero* or *one*.
*τ*	Total number of bit position set to *one* in *π*_*i*_, irrespective of their location.
$B_{kw}$	Data structure consisting of 〈*π*_0_, *π*_1_ … *π*_*n*_〉 along with corresponding 〈*τ*_0_, *τ*_1_ … *τ*_*n*_〉.
C	Search criteria containing a list of *j* search words (*sw*_0_, *sw*_1_ … *sw*_*j*_).
*ρ*_*i*_	Bloom filter encoding $sw_{i}\in C$.
H	A family of hash functions which are used to encode $kw_{i}\in I$ as *π*_*i*_ and $sw_{i}\in C$ as *ρ*_*i*_.
Q	A encrypted search query submitted to the cloud server. It contains 〈*ρ*_0_, *ρ*_1_ … *ρ*_*n*_〉 and *ϕ*.
*ϕ*	Threshold value to filter $\langle \pi_{0},\pi_{1}\;\ldots \;\pi_{m}\rangle \in B_{kw}$ to avoid unnecessary comparison operations between the outsourced data and search criteria.
$E_{S}$, $D_{S}$	Symmetric encryption and decryption algorithms.
*k*	Secret key of symmetric encryption algorithms. It is shared with authorized users only.
$E_{H}$, $D_{H}$	Homomorphic encryption and decryption algorithms.
*σ*_*pk*_, *σ*_*sk*_	Public and secret key pair for homomorphic encryption algorithms.
${\vec{\Delta }}_{0\;\ldots \;j\times m}$	Oblivious result of search query evaluation which is received by a user from the cloud server.

### 3.5 Preliminaries

In the following we describe Pascal Paillier (i.e., an additively homomorphic encryption) scheme used to obliviously process bloom filters. For more cryptographic details and security proof readers may refer to [23].

#### Key generation

Let *p* and *q* be two large primes and *n* = *p*.*q*. *ϕ*(*n*) denotes the euler’s totient function. λ(*n*) represents the carmicheal’s function. The product of two primes for *n* is *ϕ*(*n*) = (*p* − 1) (*q* − 1) and λ(*n*) = *lcm*(*p* − 1, *q* − 1). Over a multiplicative group of $F_{n^{2}}^{*}$, these two functions show the following properties:

$$|F_{n^{2}}^{*}|=\phi \left(n^{2}\right)=n.\phi \left(n\right)$$ (1)

and for any $\omega \in F_{n^{2}}^{*}$

$$\omega^{\phi (n)}=1\;\left(mod\;n\right)$$ (2)

$$\omega^{n\phi (n)}=1\;\left(mod\;n^{2}\right)$$ (3)

Public key PK is defined as (*n*, *g*), where *g* is an element of $Z_{n^{2}}^{*}$, and λ(*n*) represents the secret key SK.

#### Encryption

To encrypt a message $m\in Z_{n}$, randomly choose $y\;\in_{R}\;Z_{n^{2}}^{*}$, and define an encryption function $E_{H}$, such that:

$$E_{H}\;:\;Z_{n}\times Z_{n}^{*}\;\mapsto \;Z_{n^{2}}^{*}$$ (4)

$$E_{H}\left(m,y\right)=g^{m}y^{n}\left(mod\;n^{2}\right)$$ (5)

#### Decryption

To decrypt the ciphertext *c*, *L* is defined as (*u* − 1)/*n*, ∀*u* ∈ {*u*|*u* = 1(*mod* *n*)}. *c* can be decrypted by using secret key SK=λ(n), $D_{g}$ as

$$D_{H}\left(c,\lambda \left(n\right)\right)=\frac{L(c^{\lambda (n)}\;\left(mod\;n^{2}\right))}{L(g^{\lambda (n)}\;\left(mod\;n^{2}\right))}$$ (6)

#### Oblivious computation

Arithmetic addition between two ciphertexts, $c_{1}=E_{H}\left(m_{1},y_{1}\right)$ and $c_{2}=E_{H}\left(m_{2},y_{2}\right)$, is evaluated as:

$$\frac{\begin{array}{ccc} {ℰ}_{H}\left(m_{1},y_{1}\right) & = & g^{m_{1}}y_{1}{}^{n}\left(mod\;n^{2}\right)\\ {ℰ}_{H}\left(m_{2},y_{2}\right) & = & g^{m_{2}}y_{2}{}^{n}\left(mod\;n^{2}\right) \end{array}}{\begin{array}{ccc} 1-3{ℰ}_{H}\left(m_{1},y_{1}\right).{ℰ}_{H}\left(m_{2},y_{2}\right) & = & g^{m_{1}+m_{2}}{\left(y_{1}.y_{2}\right)}^{n}\left(mod\;n^{2}\right)\\ & = & {ℰ}_{H}\left(m_{1}+m_{2}\right) \end{array}}$$ (7)

## 4 Oblivious similarity based search: OS2

### 4.1 Initialization

The owner creates a shared repository on the cloud server which is used to persist outsourced data shared with authorized users. It then generates *k* for $E_{S}$ and $D_{S}$ to ensure privacy of F in an untrusted domain. To enable privacy-aware relevant data access the owner also initializes homomorphic cryptosystem by generating a key pair (*σ*_*pk*_, *σ*_*sk*_). Homomorphic public key (*σ*_*pk*_) is shared with the cloud server and authorized users. *σ*_*pk*_ facilitates cloud service provider to obviously evlauate encrypted search queries submitted by users. Homomorphic secret key (*σ*_*sk*_) is only shared with authorized users to enable them to query shared repository and access relevant outsourced data. An authorized user utilizes *σ*_*pk*_ to encrypt search queries and *σ*_*sk*_ to decipher oblivious research results. Key shared with an authorized user can be considered as a key-pair (*σ*_*pk*_, *σ*_*sk*_); where correct usage of each key is determined by the context. Initialization and sharing of cryptographic keys is only carried out once, after that involved entities can use them to evaluate encrypted search queries without compromising privacy of the outsourced data.

### 4.2 Bloom filter based secure indexing

**Fig 2:**
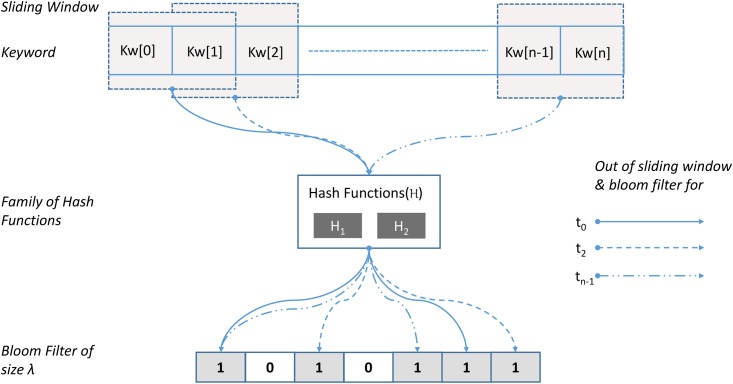
Encoding keyword *kw*[0 … *n*] as a bloom filter of length λ, with fixed window size.

Once all the necessary cryptographic primitives are initialized, the owner generates I from F, using an efficient indexing algorithm. I contains all the important keywords (*kw*_0_, *kw*_1_ … *kw*_*n*_) that constitute F. The owner can add or remove keywords from I, to ensure that authorized users can search it accordingly. After that, the owner selects publicly known H, which are then used to encode $\langle kw_{0},kw_{1}\;\ldots \;kw_{n}\rangle \in I$ as bloom filters (*π*_0_, *π*_1_…*π*_*n*_). For each $kw_{i}\in I$, the owner uses a predetermined window size to encode *kw*_*i*_ as *π*_*i*_. Each output of the sliding window is added to *π*_*i*_ by using H. Fig 2 illustrates the encoding of a keyword as a bloom filter with sliding window. Once *kw*_*i*_ is encoded to *π*_*i*_, the owner counts the number of bit locations set to one to compute *τ*_*i*_, which is used to reduce the search space. However, *τ*_*i*_ itself do not reveal any information about the actual keyword.

Since, the owner has utilized publicly known family of hash functions to generate 〈*π*_0_, *π*_1_ … *π*_*n*_〉, an entity (cloud server and malicious users) with malicious intents can compromise privacy of F, by encoding a keyword (*kw*?) of its own choice as *π*? and comparing it with 〈*π*_0_, *π*_1_ … *π*_*n*_〉. To restrain a malicious entity from deducing confidential information, each bit location of *π*_*i*_ is concealed with homomorphic encryption i.e., $E_{H}\left(\pi_{i},\sigma_{pk}\right)=\pi_{i}^{\sigma_{pk}}$. This ensures that the cloud server is able to process individual bit location in $\pi_{i}^{\sigma_{pk}}$ without compromising privacy of F. Once, $\pi_{i}^{\sigma_{pk}}$ is secured, the owner adds $\langle \pi_{0}^{\sigma_{pk}},\pi_{1}^{\sigma_{pk}}\;\ldots \;\pi_{n}^{\sigma_{pk}}\rangle$ and the corresponding 〈*τ*_0_, *τ*_1_ … *τ*_*n*_〉 to $B_{kw}$.

Since, each bit location of *π*_*i*_ is encrypted with probabilistic homomorphic encryption, malicious entities cannot differentiate between bit locations set to zero and one. Thus, restraining them from inferring confidential information by analyzing bloom filter (*π*?) of an arbitrary keyword (*kw*?) with $\langle \pi_{0}^{\sigma_{pk}},\pi_{1}^{\sigma_{pk}}\;\ldots \;\pi_{n}^{\sigma_{pk}}\rangle$.

### 4.3 Data outsourcing

Once the privacy of $B_{kw}$ is ensured through homomorphic encryption, the owner encrypts F with $E_{S}$ i.e., $E_{S}\left(F,k\right)\to F^{k}$ and outsources $F^{k},B_{kw}$ and *σ*_*pk*_ to the cloud server. After that the availability of owner is not required. Authorized users can engage in an oblivious query evaluation with the cloud server and access relevant data accordingly.

### 4.4 Query generation

User having a valid secret key (*σ*_*sk*_) can successfully query shared repository to identify outsourced data which are most relevant to its search query. To model an encrypted query for similarity based search the user defines C which contains a list of search words (*sw*_0_, *sw*_1_ … *sw*_*j*_). By using H the user then encodes $\langle sw_{0},sw_{1}\;\ldots \;sw_{j}\rangle \in C$ as 〈*ρ*_0_, *ρ*_1_ … *ρ*_*j*_〉. A predetermined windows size is used to encode *sw*_*i*_ as *ρ*_*i*_. This enables OS2 to model encrypted search queries to learn a relevance between the outsourced data and search criteria specified by a user.

Since, cloud server can exploit the bit locations of 〈*ρ*_0_, *ρ*_1_ … *ρ*_*j*_〉 to compromise privacy of $F^{k}$, the user encrypts them by using *σ*_*pk*_ shared by the owner during the initialization phase i.e., $E_{H}\left(\rho_{i},\sigma_{pk}\right)\to \rho_{i}^{\sigma_{pk}}$. Since, each bit location in *ρ*_*i*_ is encrypted probabilistically by using homomorphic encryption, cloud server cannot differentiate between two different bit location, even if both of them are set to same value.

Once, C is encoded as 〈*ρ*_0_, *ρ*_1_ … *ρ*_*j*_〉 and concealed with *σ*_*pk*_, the user transmits the encrypted search query Q to the cloud server. Q contains $\langle \rho_{0}^{\sigma_{pk}},\rho_{1}^{\sigma_{pk}}\;\ldots \;\rho_{j}^{\sigma_{pk}}\rangle$ and a threshold value (*ϕ*) which is used by the cloud server to filter $\langle \pi_{0}^{\sigma_{pk}},\pi_{1}^{\sigma_{pk}}\;\ldots \;\pi_{m}^{\sigma_{pk}}\rangle \in B_{kw}$ where *m* ≤ *n*.

### 4.5 Query evaluation

The size of bloom filter and family of hash functions that encode $\langle kw_{0},kw_{1}\;\ldots \;kw_{n}\rangle \in I$ and $\langle sw_{0},sw_{1}\;\ldots \;sw_{j}\rangle \in C$ are same. This enables OS2 to leverage cloud server to obliviously match $\langle \pi_{0}^{\sigma_{pk}},\pi_{1}^{\sigma_{pk}}\;\ldots \;\pi_{n}^{\sigma_{pk}}\rangle$ with $\langle \rho_{0}^{\sigma_{pk}},\rho_{1}^{\sigma_{pk}}\;\ldots \;\rho_{j}^{\sigma_{pk}}\rangle$. On receiving Q the cloud server filters $B_{kw}$ by using *ϕ* and identifies $\langle \pi_{0}^{\sigma_{pk}},\pi_{1}^{\sigma_{pk}}\;\ldots \;\pi_{m}^{\sigma_{pk}}\rangle$ having 〈*τ*_0_ = *ϕ*, *τ*_1_ = *ϕ* … *τ*_*m*_ = *ϕ*〉.

Once the cloud server has filtered bloom filters from $B_{kw}$ it starts the bitwise oblivious addition on $\langle \pi_{0}^{\sigma_{pk}},\pi_{1}^{\sigma_{pk}}\;\ldots \;\pi_{n}^{\sigma_{pk}}\rangle$ and $\langle \rho_{0}^{\sigma_{pk}},\rho_{1}^{\sigma_{pk}}\;\ldots \;\rho_{j}^{\sigma_{pk}}\rangle$. It computes oblivious vector ${\vec{\Delta }}_{i}$ by adding bit location of $\rho_{i}^{\sigma_{pk}}\left[x\right]$ with the corresponding bit location of $\pi_{0\ldots m}^{\sigma_{pk}}\left[x\right]$; where, *x* refers to a bit location in bloom filter, having value from 0 *to* λ. The cloud server performs the oblivious addition operation on bit locations by using *σ*_*pk*_, which is shared by the owner in the initialization phase. In total cloud server perform *j* × *m* oblivious additions.

After that cloud server replies ${\vec{\Delta }}_{0\ldots (j\times m)}$ to the user. Since, each bit location of $\pi_{i}^{\sigma_{pk}}\in Q$ and $\rho_{i}^{\sigma_{pk}}\in B_{kw}$ is probabilistically concealed, the cloud server cannot deduce any information by simply comparing them, even if bloom filters are exactly same i.e., an authorized user is searching for an arbitrary *sw*? which is exactly same as *kw*_*i*_. Besides this, oblivious addition also restrains the cloud server from learning the result of addition perform on $\rho_{i}^{\sigma_{pk}}\left[x\right]$ and $\pi_{0\ldots m}^{\sigma_{pk}}\left[x\right]$, where (0 ≤ *i* ≤ *j*) and (0 ≤ *x* ≤ λ).

### 4.6 Result post-processing

The authorized user can learn the result of encrypted search query by deciphering ${\vec{\Delta }}_{0\ldots (j\times m)}$ with *σ*_*sk*_. From deciphered bit locations it only needs to count total number of bit locations that are set to *zero* and *two*. These are the values which match with the corresponding bit location in $\langle \pi_{0}^{\sigma_{pk}},\pi_{1}^{\sigma_{pk}}\;\ldots \;\pi_{m}^{\sigma_{pk}}\rangle$. To measure the level of similarity between the encrypted search query and outsourced data it also counts the number of bit locations that are set to *one*.

Since, bloom filter encodes 〈*kw*_0_, *kw*_1_ … *kw*_*n*_〉 and 〈*sw*_0_, *sw*_1_ … *sw*_*j*_〉 as vector of *zero* and *one* the oblivious addition of a bit location can only result in *zero*, *one* and *two*. Whenever there is match between bit locations set to *one* the result is always *two*. For bit locations set to *zero* the result can only be *zero*. Since, we are dealing with *zero* and *one* values in case of a mismatch the result of oblivious addition in always be *one*.

Once user has identified matched and mismatched bit locations it uses Jaccard similarity coefficient to learn the relevance between the search criteria and outsourced data. Jaccard similarity coefficient is shown in Eq 8.

$$x=\{\begin{array}{cc} 0 & \text{matched:}\rho_{u}^{\sigma_{pk}}\left[x\right]=0,\;\pi_{v}^{\sigma_{pk}}\left[x\right]=0\\ 1 & \text{mismatched:}\;\rho_{u}^{\sigma_{pk}}\left[x\right]=0,\;\pi_{v}^{\sigma_{pk}}\left[x\right]=1\;\text{or}\;\rho_{u}^{\sigma_{pk}}\left[x\right]=1,\;\pi_{v}^{\sigma_{pk}}\left[x\right]=0\\ 2 & \text{matched:}\rho_{u}^{\sigma_{pk}}\left[x\right]=1,\;\pi_{v}^{\sigma_{pk}}\left[x\right]=1 \end{array}$$ (8)

where *x* is a bit location in a bloom filter having value 0 ≤ *x* ≤ λ. *u* and *v* are bloom filters from encrypted search query and index having 0 ≤ *u* ≤ *j* and 0 ≤ *v* ≤ *m* respectively.

$$sim\left(\rho_{u}^{\sigma_{pk}},\pi_{v}^{\sigma_{pk}}\right)=\frac{\begin{array}{c} \text{total}\;\text{number}\;\text{of}\;\text{bits}\;\text{set}\;\text{to}\;\text{zero}\\ +\\ \text{total}\;\text{number}\;\text{of}\;\text{bits}\;\text{set}\;\text{to}\;\text{one} \end{array}}{\lambda }$$ (9)

where *sim*(.) is Jaccard similarity coefficient.

With Jaccard similarity coefficient the user can identify how closely search query matches with the outsourced data. Since, the user has learned the matched and mismatched bit locations it can also apply other similarly measures according to its needs, dice co-efficient, cosine measure, to name a few.

## 5 Implementation

The proposed system of similarity based encrypted data search is implemented as a cloud service and depktop client application. Cloud service is deployed on Google App Engine [44], it is mainly responsible for persisting encrypted bloom filters ($\pi_{0}^{\sigma_{pk}},\pi_{1}^{\sigma_{pk}}\;\ldots \;\pi_{n}^{\sigma_{pk}}$) and obliviously evaluating the search queries (Q). Depktop client application is utilized to generate inverted index (I) from the data (F) before it can be outsourced to a public cloud storage service. It is also responsible for modeling encrypted search criteria $\left(\rho_{0}^{\sigma_{pk}},\rho_{1}^{\sigma_{pk}}\;\ldots \;\rho_{j}^{\sigma_{pk}}\right)$ and post process the query evaluation results (${\vec{\Delta }}_{0\ldots (j\times m)}$).

To generate inverted index we utilize Apache Lucene [45], a high performance, full-featured text search engine library. We utilize open source implementation of bloom filter [46] to encode inverted index entries ($kw_{0},kw_{1}\;\ldots \;kw_{n}\in I$) as bit strings of 0 and 1 i.e., *π*_0_, *π*_1_ … *π*_*n*_, generated by using a sliding window method illustrated in Fig 2. For our implementation we use sliding window of size two to encode inverted index entries as bloom filters. We observe that sliding window of size two is more resilient to typographical errors. This is because with sliding window size two a single typographical error is encoded twice at *t*_*l*_ and *t*_*m*_ (where *l* < *m*). For a higher value a misplaced character is encoded *g* times, where *g* is the size of sliding window.

For oblivious evaluation of search queries in an untrusted domain we utilize Pascal Paillier cryptosystem. Bloom filter bit locations for 〈*π*_0_, *π*_1_ … *π*_*n*_〉 and 〈*ρ*_0_, *ρ*_1_ … *ρ*_*m*_〉 are encrypted with secret key (*σ*_*sk*_) of Pascal Paillier cryptosystem. Whereas, set bit location counter (*τ*) is stored in plain form to ensure that cloud server can retrieve relevant encrypted bloom filters. We utilize App Engine Datastore to persist $\langle \pi_{0}^{\sigma_{pk}},\pi_{1}^{\sigma_{pk}}\;\ldots \;\pi_{m}^{\sigma_{pk}}\rangle$ and *τ*. On each search request cloud server filter $\langle \pi_{0}^{\sigma_{pk}},\pi_{1}^{\sigma_{pk}}\;\ldots \;\pi_{n}^{\sigma_{pk}}\rangle$ based on threshold value *ϕ*, and perform homomorphic addition between $\langle \pi_{0}^{\sigma_{pk}},\pi_{1}^{\sigma_{pk}}\;\ldots \;\pi_{m}^{\sigma_{pk}}\rangle$ and $\langle \rho_{0}^{\sigma_{pk}},\rho_{1}^{\sigma_{pk}}\;\ldots \;\rho_{j}^{\sigma_{pk}}\rangle$ by using *σ*_*pk*_ and replies ${\vec{\Delta }}_{0\ldots (j\times m)}$ to the client application.

## 6 Evaluation

The efficacy of our proposed similarity data search over encrypted data is tested by evaluating on desktop client application and Google App Engine cloud service. The client application and cloud service are implemented in Java using jdk 1.7.0. We use 64-bit Windows 7 machine having 3.40 GHz Intel Xeon processor and 8.0 GB main memory. Cloud service is tested on F4 and F4_G1 front-end instance classes having processing and main memory capacity as (1.2 GHz, 0.25 GB) and (2.4 GHz, 1.0 GB) respectively.

For evaluation we consider the time required to compute secure probabilistic data structure and execution overhead of oblivious comparison in Google App Engine. In the following evaluation the client application is utilized to generate secure bloom filters from the inverted index. It is also responsible for post processing the oblivious results which are replied by the cloud server. Result post processing is used to learn relevance between the search criteria and encrypted bloom filters persisted by App Engine Datastore. For cloud service, we consider the time required to obliviously add corresponding bit locations of secure bloom filters which encodes the search criteria and inverted index.

**Fig 3:**
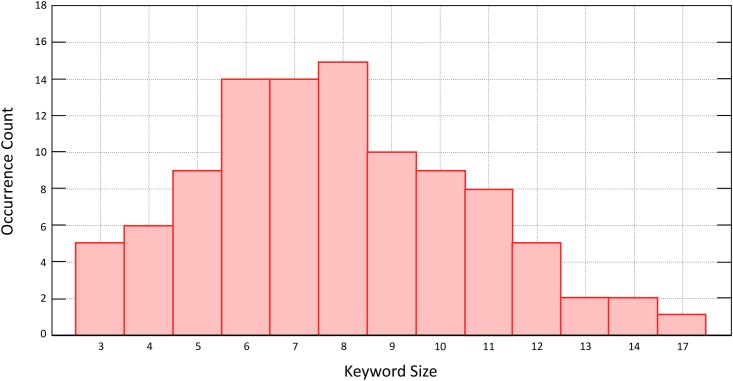
Keyword length distribution.

In this evaluation we use randomly generated English keywords. Fig 3 shows the distribution of keywords use to generate encrypted index entries $\langle \pi_{0}^{\sigma_{pk}},\pi_{1}^{\sigma_{pk}}\;\ldots \;\pi_{n}^{\sigma_{pk}}\rangle \in B_{kw}$.

### 6.1 Secure bloom filter modeling and result post processing

For secure bloom filter modeling we utilize windows size of 2. Each keyword of length *n* is divided into *n* − 1 chunks, where every chunk is encoded as bloom filter entry. For every keyword we compute a single bloom filter which is populated with *n* − 1 entries. Once keyword is encoded as a bloom filter, we count the total number of bit locations which are set to 1. After that entire bloom filter is encrypted using Pascal Paillier cryptosystem. Key size of 256 bits is utilized to encrypt the bloom filter (the proposed methodology can be extended to any key size based on security and computational requirements). Since Pascal Paillier is semantically secure, the encryption of every bit location in bloom filter resulted in a different value. This is because for each bit location Pascal Paillier utilizes a different random value *r* during the encryption process.

Post processing of oblivious results depends on threshold value used for selective retrieval of index entries at the cloud server. For this evaluation we utilize threshold value of 2. This ensure that search criteria is only compared with index entries having total set bit locations within the range *τ* ± 2. Post processing of oblivious results comprise of two steps. First, response of cloud server is deciphered by using Pascal Paillier i.e., every single bit location of oblivious result is decrypted. At this step similarity between the criteria and encrypted index is identified by learning deciphered values. 0 *and* 2 are regarded as matched; whereas, 1 is considered as a mismatched, see Eq 8. Second, once matched and mismatched values are identify we compute the similarity measure. For this evaluation we utilize Jaccard Similarity (see Eq 9) measure.

**Fig 4:**
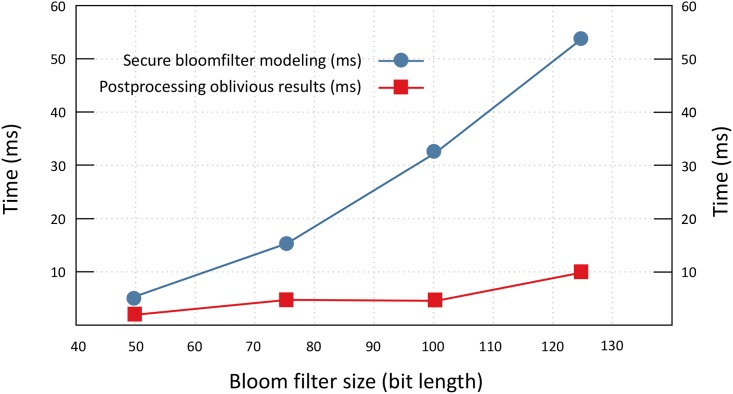
Secure bloomfilter modeling and oblivious result post processing time (sec).

Fig 4 illustrates the time required to model secure bloom filter along with the execution overhead of result post processing. For this evaluation we utilized bloom filter having 50, 75, 100, *and* 125 bit length and 1, 2, 3 *and* 4 hash functions respectively. These hash functions are utilized to set bit locations in a bloom filter. Thus the output of sliding windows (i.e., chunk) is encoded *k* times, where *k* is the total number of hash functions used to populate bloom filter. Secure bloom filter modeling shows the linear increase in execution time. With the increase in bloom filter size we also increased the number of hash functions to set respective bit locations. Since, every bit location in bloom filter is encrypted regardless of its values (0 *or* 1) the increase is execution time is mainly because of increase number of bit locations.

We utilize threshold value to avoid comparing search criteria with every index entry. Execution overhead shown in Fig 4 is mainly effected by the number of index entries having set bit locations within the range of threshold *τ* ± 2.

### 6.2 Oblivious result processing

Oblivious processing of search criteria is comprised of two steps. In the first step encrypted index entries are retrieved from the App Engine Datastore. The entries are retrieved according to the threshold value (*τ* ± 2). Once all relevant index entries are retrieved in the second step the cloud server performs homomorphic addition operation on corresponding bit locations of search criteria and encrypted index. The homomorphic addition operation obliviously results in 0, 1 *or* 2, see Eq 8.

**Fig 5:**
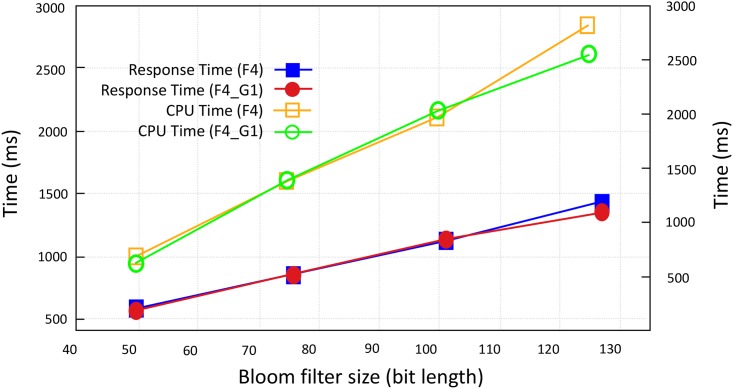
Execution overhead of oblivious evaluation of search criteria on Google App Engine (sec).

Fig 5 shows the time required to obliviously add encrypted bloom filters of search criteria and index entry. In this evaluation we ignore the time required to retrieve index entries since it is proportional to size of index. We utilized Google App Engine Frontend classed of *F*4 and *F*4_1*G*. Encrypted search criteria is oblivious added to 25 bloom filters. We evaluated execution overhead for bloom filter having 50, 75, 100, *and* 125 bit length. Fig 5 shows the response time (*ms*) and CPU Time *cpu*_*ms*. Time required to complete the oblivious addition and transmit oblivious result to the user is measured as *ms* whereas, estimated CPU cycles that can be performed by a 1.2 GHz Intel x86 processor in that amount of time [47].

## 7 Security analysis

This section presents security analysis of OS2. The analysis will focus on the capabilities of malicious cloud server and users to infer confidential information from the oblivious matching of bloom filters i.e., index entries and search queries modelled as bloom filters. Particularly, we will focus on cloud server’s advantage in exploiting oblivious matching of bloom filters bit locations. For malicious users, we will focus on users’ abilities to post unauthorized search queries and learn useful information by post-processing the search results.

The proposed methodology utilizes bloom filters and homomorphic encryption to realize encrypted data search which is capable of supporting typographical errors or misspelled search criteria. As from the descriptive details of OS2, bloom filters are used to store output of sliding window over a particular keyword (i.e., index entry and user defined search criteria). Bits locations are then used to identify similarities between index entries and search criteria. Homomorphic encryption is employed by OS2 to ensure only authorized users (i.e., users having public and secret key pair) are able to post encrypted search queries and successfully decipher the response through post-processing. OS2 also utilizes symmetric encryption to encrypt outsourced data—however, the main focus of OS2 is to facilitate similarity based search through oblivious evaluation of search queries. For the security analysis of homomorphic and symmetric encryptions, readers can refer to [23] and [48] respectively. In the following, we examine the capabilities of malicious cloud server and users to directly or indirectly infer confidential information from the oblivious processing of bloom filters.

### 7.1 Malicious cloud server

Instead of relying on a trusted third party OS2 utilizes the computational power and storage facility of a cloud server to execute search queries. The cloud server uses encrypted index $B_{kw}$ comprising of encrypted bloom filter bit locations $\langle \pi_{0}^{\sigma_{pk}},\pi_{1}^{\sigma_{pk}}\;\ldots \;\pi_{n}^{\sigma_{pk}}\rangle$ and for each bloom filter a count of bit locations set to one 〈*τ*_0_, *τ*_1_ … *τ*_*n*_〉, to process search requests.

To compromise privacy of the outsourced data, the cloud server needs to decipher the outsourced data $F^{k}$. The computational complexity to decipher $F^{k}$ is equivalent to that of symmetric encryption [48] as *k* is never shared by the data owner. The cloud server is also responsible for storing encrypted index $B_{kw}$ and evaluating encrypted search queries Q submitted in the form of $\langle \rho_{0}^{\sigma_{pk}},\rho_{1}^{\sigma_{pk}}\;\ldots \;\rho_{j}^{\sigma_{pk}}\rangle$ and a threshold *ϕ* used to narrow down the search space. To decipher the encrypted index and infer useful information from oblivious matching the cloud server needs the homomorphic encryption secret key *σ*_*sk*_. Only authorized users have access to *σ*_*sk*_ as it is distributed by the data owner during the initialization phase (although not explicitly mentioned *k* can be distributed during the initialization phase without requiring any modification to OS2, besides the encryption of *k* with authorized users’ public keys). Thus, for a cloud server the computational complexity to infer useful information from the oblivious matching of bloom filter bit locations is equivalent to that of Pascal Paillier cryptosystem [23].

### 7.2 Malicious users

OS2 enables authorized users to successfully learn from the response of the cloud server. A authorized user utilizes secret key (*σ*_*sk*_) to decipher the result of query evaluation (${\vec{\Delta }}_{0\ldots (j\times m)}$). To compromise privacy of the outsourced data $F^{k}$ and encrypted index $B_{kw}$, a malicious user would need the secret keys i.e., *k* to decrypt $F^{k}$ using symmetric encryption, and *σ*_*sk*_ to decipher ${\vec{\Delta }}_{0\ldots (j\times m)}$. Since, *σ*_*sk*_ is only shared with authorized users during the initialization phase, a malicious user cannot decipher the result of a search query. Thus, the computational complexity of successfully inferring useful information is equivalent to that of Pascal Paillier cryptosystem [23].

Although a malicious cannot successfully infer any useful information from the response of a cloud server, it can post unauthorized search queries. This is mainly due to the fact that search queries are encrypted with homomorphic encryption public key *σ*_*pk*_. Any malicious user having access to *σ*_*pk*_ can post search queries; however, *σ*_*sk*_ is required to decipher the search results. As *σ*_*sk*_ is only shared with authorized users, a malicious user would not be able to successfully infer any useful information. This can be regarded as unsuccessfully attempt by a malicious user as nothing more than the original keyword used to model the search query can be learned. To restrain malicious users from posting unsuccessful search queries, access control policies can be utilized which are beyond the scope and main objectives of OS2.

## 8 Conclusion and future work

In data driven applications (or services) data accessibility plays an important role to access and consume desired data contents by using search queries. However, the capability of relevant data access is significantly reduced to merely exact matching when user tries to securely search encrypted data persisted in an untrusted domain. This is because conventional search over encrypted data methodologies are mainly designed to ensure confidentiality of search queries and do not consider user’s search experience. In this work we presented oblivious similarity based search for encrypted data (OS2). It leveraged authorized subscriber(s) of a public cloud storage service to obliviously learn relevance between user defined encrypted search criteria and outsourced data. Unlike conventional methodologies which mainly rely on computationally intensive private matching protocol or trapdoor based cryptography to search encrypted data, OS2 utilized homomorphic addition over secure probabilistic data structure to learn similarity measure between search query and encrypted data. With OS2 search queries were evaluated within the untrusted domain of cloud service provider without relying on trusted/semi trusted entities. This enabled us to fully utilize computational facilities of public cloud service provider. Evaluation of OS2 on Google App Engine highlighted the fact that it exerted amicable execution load on involved entities; whereas, offloading computational load on public cloud without compromising confidentiality and privacy of search query and outsourced data.

With this research we have demonstrated that it is possible to obliviously search encrypted data with search queries which are resilient to typographical errors. Another interesting yet challenging direction for similarity based encrypted data search is contextually informed privacy-aware search. Sensing and actuating devices in internet-of-things can benefits from it by accessing private and confidential sensed data within a given context.
